# The Role of Protein Kinases in the Management of Oncological Diseases by Acting on Ferroptotic Pathways

**DOI:** 10.3390/ijms27062673

**Published:** 2026-03-14

**Authors:** Valentina Folgiero, Matteo Caforio

**Affiliations:** Department of Onco-Hematology and Cell and Gene Therapy, Bambino Gesù Children’s Hospital, IRCCS, 00146 Rome, Italy

**Keywords:** kinases, ferroptosis, cancer, lipid peroxidation, immunotherapy

## Abstract

Ferroptosis is a recently discovered form of cell death, driven by membrane lipid peroxidation with the contribution of intracellular iron. In recent years, many researchers have discovered the involvement of ferroptotic mechanisms in the etiology of various diseases, including several forms of cancer. Different points in the ferroptotic pathway can be crucial for arising or sustained pathologies, given the contribution of numerous molecular mechanisms concerning membrane channels, several proteins, enzymes, and also kinases. The latter, in particular, seem to be very important in the control of ferroptosis in different manners depending on the pathology. Therefore, many articles in recent years have described how the pathways that involve kinases can determine, control, or alter the physiological ferroptotic contribution. Interestingly, in a tumoral context, oncogenes and tumor suppressor activity affect the correct ferroptotic process directly or indirectly promoted by abnormal kinase activity. Expanding the understanding of how kinases contribute to tumorigenesis by altering ferroptosis mechanisms may provide important insights to improve current anticancer therapies. Furthermore, new data have indicated how kinase-dependent ferroptotic activity may influence the efficacy of immunotherapy. Since one of the major obstacles to this promising anticancer therapy concerns the resistance induced by cancer cells, finding new targets, such as kinases, to improve ferroptosis in tumor cells could open an intriguing door to enhancing immunotherapy and overcoming the current obstacle.

## 1. Introduction

Ferroptosis is a recently discovered form of regulated cell death, which, along with elevated iron ion levels, is driven by the peroxidative damage of polyunsaturated-fatty-acid-containing phospholipids in cellular membranes [1,2]. Ferroptosis is triggered by the inactivation of glutathione (GSH)-dependent cellular antioxidant defenses, leading to the accumulation of toxic lipid-reactive oxygen species (L-ROS) [2,3].

Iron accumulation is linked to ferroptosis through two mechanisms: on the one hand, it promotes the excessive production of ROS via the Fenton reaction, which increases lipid oxidative stress levels [4,5,6]. On the other hand, iron participates in the activation of lipoxygenases (LOX), which are iron-containing enzymes that promote the generation of lipid ROS (e.g., lipid peroxides) [7]. Increased lipid peroxidation results in cellular and mitochondrial membrane damage and induces ferroptosis [8]. Circulating Fe^3+^ enters the cells via the transferrin/transferrin receptor-1 (TF/TFR-1) transport system, where it is reduced to Fe^2+^ and transferred into the cytoplasm, inducing physiological and biochemical processes, including DNA biosynthesis, oxygen transport, and the regulation of metabolic pathways [9]. When iron-binding complexes reach saturation, the excess Fe^2+^ accumulates in an unstable iron pool. The Fe^2+^ present in the iron pool participates in the Fenton reaction to generate ROS, mainly hydroxyl radicals. This leads to membrane lipid peroxidation and ultimately ferroptosis [5]. Another important member controlling ferroptosis through GSH is GPX4, which serves as a vital substrate for this enzyme. Its activity is dependent on the System Xc-, constituted by heterodimers, which comprise Solute Carrier Family 7 Member 11 (SLC7A11) and Solute Carrier Family 3 Member 2 (SLC3A2) [10] that exchange extracellular cystine uptake and intracellular glutamate efflux in a 1:1 ratio. GSH is produced from intracellular cystine, where it allows GPX4 to convert phospholipid peroxides to phospholipids, repressing ferroptosis [11].

### 1.1. Kinases Involved in the Ferroptosis Mechanism

#### 1.1.1. MAPKs and AMPKs

The dysregulation of the mitogen-activated protein kinase (MAPK) and AMP-activated protein kinase (AMPK) pathways, which represent the regulation of oxidative and energy stress, is critical for ferroptosis. The MAPK family involves extracellular signal-regulated kinase (ERK), c-Jun N-terminal kinase (JNK) and p38 mitogen-activated protein kinase (p38 MAPK), exerting a critical role in cell growth, differentiation, apoptosis and the response to oxidative stress, the last one connecting MAPK’s activity to ferroptosis.

AMPK, under physiological conditions, exerts a pivotal role in processes such as cell growth, autophagy and apoptosis. But its connection with ferroptosis is due to its function as a sensor of cellular energy when the accumulation of lipid peroxides induces energy stress [1]. The activation of AMPK results in the modulation of autophagy, mTORC1 signaling, p53 activation, cystine uptake and iron metabolism. In addition, it has been recently discovered that MAPK can activate the LKB1-AMPK signaling pathway, making their mechanism of action more complicated [1,10,12].

Among the members of the AMPK family, Salt-inducible kinase 1 (SIK1) is a key regulator of cellular metabolism, growth and vascular remodeling [13]. It regulates the progression of various cancer types, functioning as a tumor suppressor or oncogene in diverse contexts [14,15,16]. SIK1 expression is regulated by external factors that are constitutively expressed. It is abundant in nervous tissue, adipose tissue and the adrenal cortex [16,17,18]. It is involved in numerous physiological processes dedicated to energy production, including gluconeogenesis [19,20] and lipid metabolism [21,22]. Moreover, SIK1 has been found to be involved in apoptosis [23] and in the regulation of circadian rhythms and sleep [24,25]. In most tumor types, SIK1 has been shown to inhibit proliferation, invasion and migration [16,26], albeit sometimes through overexpression and, in other tumor contexts, through its downregulation, a behavior that remains to be elucidated. Its role as regulator of ferroptosis resistance in pancreatic ductal adenocarcinoma (PDAC) through the HDAC5-STAT6-SLC7A11 axis [27] has recently been discovered.

#### 1.1.2. PKCβII

PKCβII is a member of the protein kinase C (PKC) family of Ser/Thr kinases and has strong activity in several signaling pathways [28], including proliferation, polarity, motility and differentiation. When localized in the nucleus, PKC enzymes control the gene expression of transcription factors involved in the differentiation of pluripotent stem cells toward epithelial, mesenchymal and hemopoietic lineages. Under pathological conditions such as cancer, the regulation of transcription factors is aberrantly controlled, inducing invasiveness, stemness and tumor microenvironment dysregulation [29]. The connection between PKCβII and ferroptosis arises from its ability to function as a sensor of lipid peroxidation through the activation of Acyl-CoA synthetase long-chain family member 4 (ACSL4), a lipid metabolism enzyme required for lipid peroxidation, improving immunotherapy in melanoma [30].

#### 1.1.3. CDK7

Cyclin-Dependent Kinases (CDKs) are mainly known as the kinases involved in cell cycle phases in association with activating partners, the cyclins [31]. It is now evident that a specific subgroup of this family exerts different functions beyond cell cycle progression [32]. Indeed, it has recently been shown that CDK7 is involved in the regulation of stem cell-like properties in Esophageal Squamous Cell Carcinoma (ESCC). In particular, CDK7 participates in the regulation of esophageal CSC hallmarks and plays a catalytic role, by promoting the transfer of phosphate groups from ATP to their targeted substrates. Indeed, CDK7 phosphorylates Yes-associated-protein (YAP) at its S127 and S397 sites and induces protein expression through physical interaction in the nucleus. Because lactate dehydrogenase-D (LDHD) is highly enriched in ESCC and is responsible for stemness-associated hallmarks, the possibility that the CK7-YAP-LDHD axis could sustain stem-like potential in ESCC was tested. This evidence was confirmed by demonstrating that the decrease in D-lactate and the increase in pyruvate due to the CDK7-YAP-LDHD axis in mitochondria are necessary to maintain the self-renewal and proliferative potential in ESCC CSCs. Furthermore, overexpression of the axis decreases lipid ROS generation, reducing the ferroptosis mechanism [33].

#### 1.1.4. PLK1

Polo-Like Kinase 1 (PLK1) plays a critical role in several phases of mitosis [34,35,36] and is highly expressed in tumors of various origins but absent in surrounding normal tissues [37,38,39]. PLK1 is found primarily in the cytoplasm and localizes to the nucleus when its levels increase during the G2/M phase. The C-terminal polo-box domain (PBD) of PLK1 in mitotic cells allows its localization to the centrosome, kinetochore, spindle midzone, centromere and post-mitotic bridge [35]. Post-translational modifications of PLK1 and interactions with key partner proteins regulate its kinase activity, but an additional function is exerted by PBD, which acts as both an autoinhibitory and a subcellular localization domain [40]. PLK1 inhibition can selectively kill cancer cells dependent on PLK1 overexpression [39,41,42]. In particular, in metastatic colorectal cancer (mCRC) harboring the *BRAF* mutation V600E, BRAFi ± Epidermal Growth Factor Receptor inhibitor (EGFRi) therapy induces upregulated GPX4 expression, which antagonizes lipid peroxidation and ferroptosis via ROS. Through integrated proteomics and phosphoproteomics analyses, it was revealed that CBX8 phosphorylation promotes *GPX4* transcription in response to BRAFi ± EGFRi, repressing ferroptosis [43].

#### 1.1.5. PI3K-AKT-mTOR

Aberrant activation of the phosphatidylinositol-3 kinase-AKT-mammalian target of Rapamicin (PI3K-AKT-mTOR) pathway occurs in approximately 50% of tumors and represents the most commonly activated pathway in human cancer, frequently causing resistance to treatments [44,45,46]. Aberrant expression or mutation of many components in this pathway is related to human oncogenesis [47], such as mutation or amplification of *PIK3CA*, the gene encoding the catalytic subunit of PI3K, p110a or inactivation of the tumor suppressor phosphatase and TENsin homolog (PTEN). Sterol regulatory element-binding protein-1 (SREB-1) is a member of the SREBPs family of transcription factors belonging to the basic helix–loop–helix leucine zipper family that are synthesized as precursors and bound to the endoplasmic reticulum membrane [48]. When induced by specific signals, they translocate to the Golgi apparatus where they are cleaved, releasing the mature form, which can translocate to the nucleus and activate target gene expression. Since SREBP-1 can regulate carbohydrate metabolism in addition to hepatic de novo lipogenesis, a reduction in its activity has therapeutic potential to reduce hepatic lipid accumulation and improve insulin sensitivity by blocking gluconeogenesis and hepatic glucose output [48], influencing PI3K-AKT-mTOR inhibition of ferroptosis. Indeed, activation of the PI3K-AKT-mTORC1 signaling pathway enables cancer cells to develop resistance to ferroptosis through the upregulation of downstream SREBP1-mediated lipogenesis in breast and prostate cancer [49].

#### 1.1.6. SGK2

Serum and glucocorticoid kinases (SGKs), members of the AGC family of serine/threonine kinases, share the highest homology with the AKT family, having a similar domain structure and high sequence identity in the catalytic domain [50,51,52]. SGK-2 has two variants, named alpha and beta, which differ in their amino-terminus and have been associated with prostate cancer metastasis [53]. The mechanism involves the regulation of ferroptosis through the upregulation of the ferroptosis inhibitor GPX4.

#### 1.1.7. TBK1

The serine/threonine protein kinase TBK1, a member of the IκB kinase (IKK) family, promotes type I interferon (IFN) and modulates NF-κB signaling in order to regulate the innate immune response [54,55]. In addition, its involvement in the regulation of cell proliferation, survival, cell death, signaling transduction and metabolism has been demonstrated [56,57], while its dysregulation is strictly related to cancer development [58]. Given the role of ferroptosis in regulating cell death, its induction has been well recognized as an effective antitumor strategy. Sorafenib has recently been identified as a ferroptosis inducer in hepatocellular carcinoma (HCC) cell lines [6], and it is also known that ferroptosis prevents sorafenib resistance in HCC [59]. TBK1 suppresses ferroptosis by promoting Nrf2 stability. Tiliroside (a natural inhibitor with a still unknown target)-dependent TBK1 inhibition induces ferroptosis, promoting NRF 2 ubiquitination and degradation, potentiating the efficacy of sorafenib [55].

#### 1.1.8. Nrf2: An Important Transcription Factor Involved in Ferroptosis Regulated by Several Kinases

Nrf2 is a transcription factor that regulates the expression of a large number of genes involved in oxidative stress, redox homeostasis, metabolism, DNA repair, cell survival and proliferation [60]. In cancer, it sustains two pivotal functions, one in counteracting tumor initiation and the other in promoting cancer progression and resistance to therapy [61]. When upregulated in cancer, it is able to transactivate several genes involved in iron metabolism, ROS detoxification and GSH metabolism [62]. Since more than 60% of glioblastoma (GBM) cases show aberrant activation of tyrosine kinase family members, in particular downstream Src kinase signaling, this mechanism has been analyzed, resulting in overexpression and able to induce Nrf2 localization to the nucleus. Here, it transcribes genes related to oxidative stress responses and ferroptosis regulation. In meningioma cells, the transcription factor MEF2C upregulates the expression of *Nrf2* and E-cadherin, thereby inhibiting Erastin-induced ferroptosis [63,64]. In other tumor contexts, the role of Nrf2 is supported by the kinase AURKA, which, through its upregulation, sustains the malignity of meningioma and the expression of anti-ferroptosis genes [65]. Furthermore, other recent studies have identified Nrf2 as a direct or indirect target of other important kinases, such as TBK1 and MAPKs, in different tumor contexts [66].

## 2. Main Text

The involvement of ferroptotic pathways in cancer development has recently been described. However, not all aspects have been clarified, and new data have emerged, revealing new players in ferroptosis-dependent cancer development. In this field, different kinases play different roles, depending on the context, resulting in a different response in the modulation of ferroptosis. Many kinases act on the main anti-ferroptotic axis, which includes SLC7A11, GSH, and the enzyme GPX4. In this context, although some kinases can directly target these factors, their modulation can sometimes be indirect, starting further upstream [14,43,53]. Another level of ferroptosis regulation under kinase control targets Nrf2 and KEAP1, whose interaction is crucial for regulating Nrf2 transcriptional activity [60,63]. Other factors may also be involved, albeit less frequently, but with significant consequences for ferroptotic mechanisms. These may include pathways that alter the type of fatty acids present in the membrane, determining a greater or lower sensitivity to ferroptosis [28,49]. Certain metabolites can also influence ferroptosis regulation, as occurs with the presence of pyruvate, whose levels can be increased through the activation of enzymes regulated by certain kinases [33]. A slightly separate parenthesis concerns MAPKs, which can be controlled by different stimuli and can, therefore, be involved in ferroptotic modulation in either a positive or negative sense, intervening in different pathways [67] (Table 1).

### 2.1. Kinases That Affect System Xc-/GSH/GPX4 Pathway

#### 2.1.1. Ferroptosis Control by PLK1-CBX8-GPX4 Axis in BRAFV600E Colorectal Cancer

**Figure 1:**
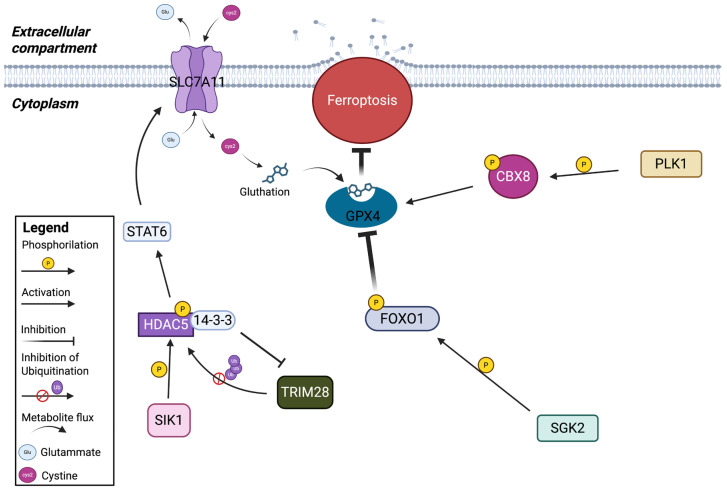
Kinases involved in the regulation of ferroptosis acting on SLC7A11/GSH/GPX4 axis.

GPX4 is the most powerful factor capable of protecting the cells from ferroptosis, thanks to its ability to neutralize ROS and reduce oxidative stress. Its expression has also been linked to a poor prognosis in patients with colorectal cancer (CRC) carrying mutation on *BRAF* (BRAFV600E) [68]. Interestingly, treatment with BRAF and EGFR inhibitors increases ROS production, but a recent study demonstrated that inhibition of BRAF (BRAFi) and EGFR (EGFRi) can increase GPX4 levels in CRC cells. These cells are resistant to BRAFi, because increased ROS production is neutralized by the cells through GPX4 activity. In this context, they demonstrated that *CBX8*, a gene associated with poor prognosis, may trigger ferroptosis by regulating *GPX4* expression in BRAFV600E CRC. Indeed, the authors observed the CBX8 binding site on the promoter of *GPX4*. In particular, the phosphorylation of CBX8 is crucial for promoting *GPX4* expression, and the authors identified PLK1 as a key actor in this cascade, as it translocates into the nucleus and phosphorylates CBX8 on the Ser265 residue under BRAFi treatment, with or without EGFRi. Targeting PLK1 induces the inhibition of *GPX4* and induces the activation of ferroptosis mechanisms in vitro, in vivo and in organoid models. In summary, they demonstrated a PLK1–CBX8–GPX4 signaling axis that relays the ferroptosis mechanism of therapeutic resistance (Figure 1). For this reason, PLK1 inhibition could be a strategy to overcome BRAFi resistance in BRAFV600E CRC [43].

#### 2.1.2. Ferroptotis Inhibition by SGK2 via Upregulating GPX4 in Prostate Cancer

Another context in which the regulation of GPX4 plays a pivotal role concerns prostate cancer (PC) metastasis. Here, the role of SGK2, a member of the SGK kinases family, already studied for its role in bladder, colon and kidney cancer [69], has been deeply studied. The authors demonstrated that SGK2 expression induces the nuclear exclusion of FOXO1, whose function is to negatively regulate *GPX4*, while FOXO1 overexpression reverses the increase in GPX4 protein levels induced by SGK2 overexpression (Figure 1). They found that SGK2 phosphorylates FOXO1 at three specific residues, excluding it from the nucleus and, thus, leading to the upregulation of *GPX4* expression. In this context, protein kinase B (PKB) may also play a role in phosphorylating FOXO1 at other specific sites in order to reinforce the nuclear exclusion. This mechanism ultimately promotes PC metastasis, as GPX4 expression protects against ferroptosis, leading to increased resistance. These findings may also be translated into new therapies for the treatment and prevention of PC at metastatic stage [53].

#### 2.1.3. SIK1 Promotes Ferroptosis Resistance in Pancreatic Cancer

SLC7A11, as a regulator of intracellular cystine levels, is highly regulated in cells. Kinases may also play a key role in this regulation, becoming an important factor in ferroptosis control. Recently, SIK1, a member of the AMPK family, was identified as a regulator of ferroptosis resistance in pancreatic ductal adenocarcinoma (PDAC) following kinase database screening. Briefly, SIK1, through its kinase activity, phosphorylates Histone Deacetylase 5 (HDAC5), promoting its interaction with the factor 14-3-3, which is known to protect HDAC5 from the degradation mediated by TRIM28. Stabilization of HDAC5 can lead to the deacetylation of Signal Transducer and Activator of Transcription (*STAT6*), enhancing its transcriptional activity, which can upregulate SLC7A11 expression (Figure 1) and confer ferroptosis resistance in PDAC. This mechanism is specific to HDAC5 and not to other members of the HDAC family. The literature describes that HDAC5 can lead to increased iron levels, ROS production and arachidonic acid metabolism [70,71]. Additionally, Yu et al. showed that PDAC cells were more sensitive to ferroptosis under conditions of oxidative stress. SIK1 depletion was found to increase sensitivity to ferroptosis, which can be reversed by overexpression of HDAC5. They also found that phosphorylation at Ser498 of HDAC5 is critical for its stability. The E3 ubiquitin ligase Tripartite Motif 28 (TRIM 28), which mediates the ubiquitination and subsequent degradation of HDAC5, cannot exert its activity because the activity of SIK1 on HDAC5 strongly increases the affinity between HDAC5 and 14-3-3, preventing the ubiquitination of TRIM28 on HDAC5. In this way HDAC5 is stabilized and can exert its deacetylating activity on *STAT6*. STAT6 has been observed to upregulate SLC7A11 in PDAC cells, acting as an activating transcription factor capable of binding the promoter region of the *SLC7A11* gene. This study supports the emerging role of SIK1, HDAC5 and STAT6 in the ferroptotic mechanism, particularly as ferroptosis protectors. Furthermore, inhibition of STAT6 may also limit PDAC growth by deregulating other genes able to sustain proliferation [72]. Targeting the SIK1/STAT6 axis can have multiple effects in improving cancer therapies [14].

### 2.2. Kinases That Affect NRF2/KEAP1 Interaction

#### 2.2.1. Contribution of Src Tyrosine Kinase to Ferroptosis Resistance in Glioblastoma

**Figure 2:**
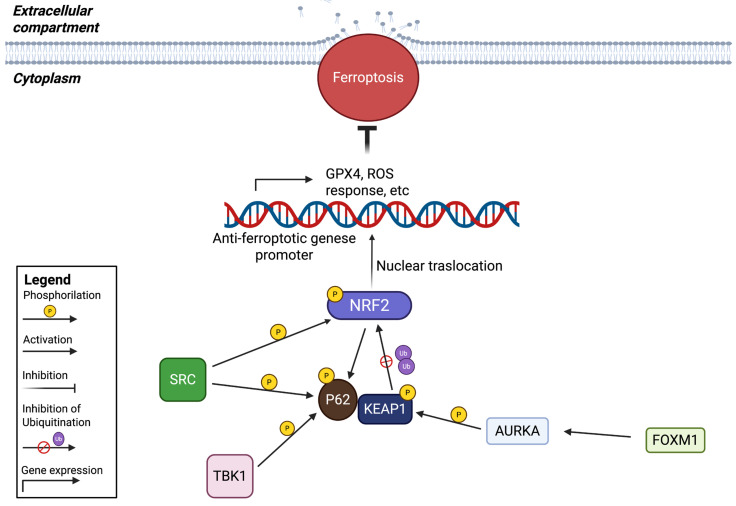
Kinases involved in the regulation of ferroptosis affecting Nrf2/KEAP1 interaction.

The mechanism by which the cells use GPX4 to avoid ferroptotic death is crucial. For this reason, *GPX4* is controlled by several transcription factors. One of the most important is Nrf2, which counteracts ferroptosis not only through GPX4 but also by transactivating several cytoprotective genes involved in iron metabolism and ROS detoxification [73]. Recently, Nrf2 has been uncovered as a new player in Src deregulation in GBM. A link has been found between the aberrant activation of Src and the inhibition of ionizing radiation-induced ferroptosis. In this case, Src, through its kinase activity, sustains the localization of Nrf2 in the nucleus, favoring the activation of genes involved in oxidative stress responses. Src can also cause the aggregation of p62 and its colocalization with the KEAP1 factor [74]. KEAP1, when Src is not activated, can interact with Nrf2, promoting competition between p62 and Nrf2 for KEAP1 binding [75]. The role of KEAP1 is to promote Nrf2 degradation; therefore, this competition has a critical regulatory role in the ferroptosis system (Figure 2). Thus, Src activity plays multiple roles in sustaining ferroptosis, both by inducing Nrf2 activation and by impeding Nrf2 degradation. Furthermore, Nrf2 can also induce *p62* expression, creating a positive feedback loop capable of amplifying the anti-ferroptotic mechanisms. It has been demonstrated that Src-dependent Nrf2 activation contributes to increasing sensitivity to ferroptosis. They evaluated the contribution to ionizing radiation therapy, demonstrating that Src-dependent Nrf2 activation may contribute, by increasing sensitivity of ferroptosis, to enhancing irradiation therapy for GBM. They provide evidence for a new link between activation of Src tyrosine kinase and the constitutive activation of Nrf2 signaling, identifying an unexpected role for phosphorylation signaling in the modulation of ferroptosis [60].

#### 2.2.2. FOXM1-AURKA-Nrf2 Axis in Erastin-Induced Ferroptosis Resistance in Meningioma

Recently, a role for AURKA has been found in the ferroptosis mechanism in meningioma. It is upregulated in high-grade contexts and facilitates malignancy. Also, in this case, the KEAP1 factor is involved, which undergoes phosphorylation via the work of AURKA [65]. Under normal circumstances, the Nrf2 protein is principally degraded, and its basal level is maintained at a low level by different E3-ubiquitin ligase complexes, involving KEAP1. The phosphorylation performed by AURKA at Thr80 of KEAP1 prevents the interaction between KEAP1 and Nrf2 (Figure 2), permitting the nuclear localization of Nrf2 and the expression of downstream anti-ferroptotic genes [65]. In addition, the authors found that FOXM1, a member of the Forkhead box (Fox) transcription factor family known for playing crucial roles in tumor development and progression [76], can positively regulate *AURKA*. They deeply investigated this aspect because, in a previous article, the same authors found that FOXM1 mRNA levels increase in meningioma compared to normal tissues, particularly in high-grade meningiomas compared to grade I tumors [77]. Therefore, this study showed that the increase in Nrf2 levels in higher-grade meningiomas depends on the overactivation of the FOM1-AURKA axis, supporting the idea that high Nrf2 expression levels can be a biomarker for higher-grade meningioma and increased resistance to ferroptosis. Through these mechanisms, FOXM1 possesses the ability to impede Erastin-induced ferroptosis, confirming other previous works that reported the inhibitory role of FOXM1 in ferroptosis. The link with AURKA has already been found in breast cancer stem cells, in which FOXM1 transcriptionally activates *AURKA* expression [78]. Similarly, they confirmed that FOXM1 acts as a transcription factor responsible for the upregulation of *AURKA* in meningioma, inhibiting Erastin-induced ferroptosis via the activation of Nrf2 and all its downstream anti-ferroptotic genes, including *SLC7A11*, ferritin heavy chain 1 (*FTH1*), Ferritin Light Chain (*FTL*), and Heme Oxygenase 1 (*HMOX1*) [62]. Interestingly, they did not observe the same results using the ferroptosis inducer RSL3 treatment, indicating that the action of the FOXM1-AURKA axis is specific to Erastin-dependent ferroptotic induction. They speculated that the use of Erastin, which is not currently used clinically for tumor treatment, could be enhanced by also targeting the FOXM1-AURKA-Nrf2 pathway [63].

#### 2.2.3. Targeting TBK1 to Induce Ferroptosis in Hepatocellular Carcinoma

Nrf2, as a strong factor involved in ferroptosis, has been deeply studied, with the aim of targeting the pathways which control it. Yang et al. studied a compound, named Tiliroside, which exhibited significant antitumoral activity in HCC and, at the same time, was able to enhance the activity of Sorafenib, a compound already approved for HCC treatment. They discovered the mechanism of action of Tiliroside: it can bind to and inhibit TBK1 activity, promoting Nrf2 ubiquitination and degradation, thereby promoting ferroptosis and improving the effect of Sorafenib. In detail, they demonstrated the capacity of Tiliroside to weaken or impede the phosphorylation of Ser349 on p62 mediated by TBK1. This action is crucial because this specific phosphorylation event at Ser349 in p62 has recently been implicated as a mediator of the interaction between p62 and KEAP1 (Figure 2). This reinforces the binding between KEAP1 and Nrf2, triggering ferroptosis and underlying how Tiliroside could represent a potential treatment option for HCC. Tiliroside-induced ferroptosis in HCC cells is strongly associated with the disruption of oxidative stress balance, as well as in the ferroptosis-related gene set. In fact, they observed a decrease in GPX4 expression, whose targeting was already tested, showing a combined effect with Sorafenib in HCC [79]. However, they also observed a decrease in two other regulatory factors of ferroptosis: FTH1 and Glucose-6-phosphate dehydrogenase (G6PD) [80,81]. Interestingly, the genes encoding these proteins are Nrf2 target genes. The evaluation of Nrf2 cellular protein levels showed a decrease concurrently with reduced Nrf2 translocation to the nucleus under Tiliroside treatment, inhibiting the transcription of its target genes. Analysis of the molecular structure found that Tiliroside directly binds to the hinge region of TBK1 with kinase activity and inhibits its activation. In this context, phosphorylation of Ser172 is critical for kinase activity. Their results indicated that the levels of phosphorylated TBK1 were significantly downregulated under Tiliroside administration, confirming its function as a TBK1 inhibitor. In summary, they found a novel target of the TBK1 kinase, which inhibits its activity, preventing the translocation of Nrf2 into the nucleus and the resulting expression of anti-ferroptotic genes. Pro-ferroptotic genes coordinate sensitivity to ferroptotic cell death in HCC cells, which may improve Sorafenib treatment [55].

### 2.3. Kinases That Modulate the Presence of Fatty Acids on the Membrane

#### 2.3.1. Involvement of PKCβII in Lipid Peroxidation Acting on ACSL4

**Figure 3:**
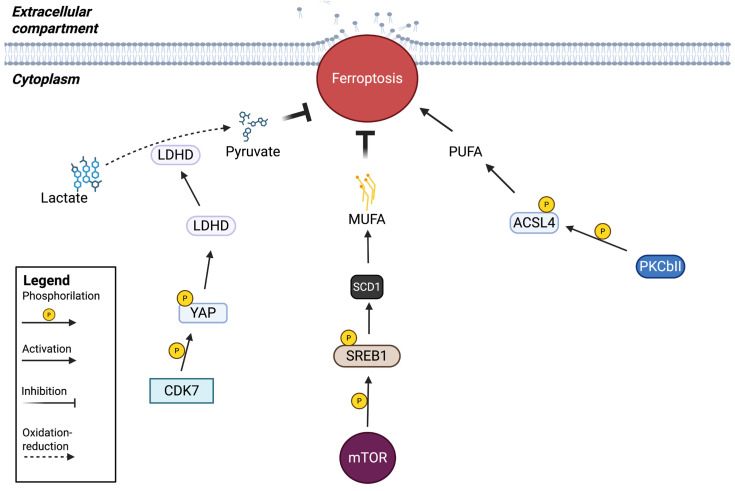
Kinases involved in the production of different types of membrane lipids (MUFA and PUFA), and other metabolites involved in ferroptosis mechanisms, such as the lactate/pyruvate transition.

PKCβII has been identified as a critical contributor to ferroptosis, amplifying lipid peroxidation through its phosphorylation of ACSL4. PKCβII, in fact, directly interacts with ACSL4 and phosphorylates it at Thr328, triggering PUFA-containing lipid biosynthesis and promoting the generation of lipid peroxidation products (Figure 3). In this way, the lipid peroxidation–PKCβII–ACSL4 axis creates a positive feedback loop that induces ferroptosis by amplifying lipid peroxidation to lethal levels. The key point discovered by the scientists in this study is the importance of this loop. Indeed, the common amount of PUFA present in the membrane may not be sufficient to reach lethal levels of lipid peroxidation within cells. Amplification of membrane lipid peroxidation induced by the PKCβII–ACSL4 positive feedback loop enhances PUFA synthesis by generating substrates for further lipid peroxidation. This amplification represents a prerequisite for ferroptosis. The phosphorylation of ACSL4 performed by PKCβII plays a pivotal role in this mechanism; in fact, experimental data have shown that a mutation at Thr328 of ACSL4 impairs ferroptosis, both in vivo and in vitro. This kind of phosphorylation increases during ferroptosis, and several data have demonstrated that ferroptosis contributes to tumor cell death, favoring cancer immunotherapy. Therefore, elucidating the pathway involved in ACSL4 activation can potentiate current cancer immunotherapy strategies. The same authors discovered that the PKCβII–ACSL4 axis affects the efficacy of cancer immunotherapy, suggesting that this pathway may represent a predictor of response to immunotherapy. Other findings confirm that attenuation of this pathway decreased the efficacy of cancer immunotherapy by inhibiting ferroptosis, suggesting that an in-depth study of this pathway could offer potential targets and strategies for ferroptosis-associated cancer therapy [28].

#### 2.3.2. PI3K-AKT-mTOR Signaling Suppresses Ferroptosis Through Lipogenesis Activation

Ferroptosis can also be regulated in an indirect way, not only through the key genes that sustain or repress it. As far as this aspect is concerned, the PI3K-AKT-mTORC1 pathway suppresses ferroptosis in cancer cells via downstream SREBP1/Sterol regulatory element-binding protein-1 (SCD1)-mediated lipogenesis. The involvement of other metabolic processes capable of controlling ferroptosis, such as glutaminolysis and the TCA cycle [82], as well as cellular metabolic pathways involving carbohydrates, lipids and amino acids [83], has been previously demonstrated. mTOR is an important modulator of cellular redox homeostasis, and the demonstrated oncogenic alterations in PI3K-AKT-mTOR signaling, one of the most frequently mutated pathways in human cancer [84], make cancer cells more resistant to ferroptosis induction. In fact, mTOR inactivation, in response to ferroptosis inducers, increases membrane lipid peroxidation. In mutant cancer cells, hyper-activation of mTORC1 alone, and not mTORC2, prevents lipid peroxidation accumulation, thus causing resistance to ferroptosis. It has been reported that mTORC1 promotes the association of p62 with KEAP1 by phosphorylating p62, leading to the degradation of KEAP1 and consequently to the accumulation of Nrf2 (Figure 3) [85]. In this way, the p62-KEAP1-Nrf2 axis can protect hepatocellular carcinoma cells from ferroptosis [86]. Nrf2 signaling is one of the factors that mediate the effect of mTOR on ferroptosis inhibition [86,87]. However, in a recent paper, researchers showed that the central lipid regulator SREBP1 [88], which is a target of mTOR [89], decreases the level of the mature form of SREBP1, and CRISPR/CAS9-dependent inhibition of *SREBP1* sensitizes cells to ferroptosis and lipid peroxidation. SREBP1 is a transcription factor, and by analyzing its target genes, the authors observed a decrease in *SCD1* gene expression following its inhibition. This gene is responsible for the protection from ferroptosis mediated by SREBP1. SCD1 is an enzyme that converts saturated fatty acids to monounsaturated fatty acids (MUFAs), and it is well known that MUFAs can inhibit ferroptosis [90], providing evidence of the anti-ferroptotic activity of SCD1. Thus, the PI3K-mTOR pathway, acting on the SREBP1 transcription factor, protects cells from ferroptosis because SCD1 can modulate lipid metabolism, supporting MUFA production rather than polyunsaturated fatty acids (PUFAs), showing anti-ferroptotic activity both in cell cultures and in mouse xenograft models [49].

### 2.4. Kinases That Increase Intracellular Metabolites

#### Role of the CDK7-YAP-LDHD Axis in Supporting Cancer Stem Cell-like Properties in ESCC

GPX4 and Nrf2 exhibit a fundamental role in the ferroptotic pathway, and their activities are often correlated, as GPX4 can be controlled by Nrf2 activity. However, other GPX4-independent mechanisms involving kinase activity may also operate, contributing to the protection of cells against ferroptosis by exerting anti-ferroptotic effects. For example, a recent paper found a role of CDK7-YAP-LDHD axis in ferroptosis defense in a cancer stem cell-like context, sustaining ESCC. Previous studies have demonstrated that the CSC population exists in ESCC [91,92]. However, the mechanisms underlying the maintenance of ESCC-CSC stemness have not been well elucidated. In this study, they revealed a novel machinery, in which the nuclear CDK7-YAP complex is responsible for maintaining the stem cell-like properties of esophageal CSCs. Indeed, they found a small-molecule inhibitor targeting CDK7 with the power to eradicate cancer stem cell properties. The kinase CDK7 can promote the CSC phenotype by increasing YAP levels, which, in turn, may enhance *LDHD* expression. LDHD is able to accelerate D-lactate catabolism in ESCC-CSCs, bringing protection against ferroptosis. It is well known, in fact, that excess D-lactate represses the level of the xCT channel and decreases *GPX4* expression, supporting the pro-ferroptotic pathway [93]. When the CDK7-YAP-LDHD axis is active, pyruvate, a catabolite of D-lactate, is generated, contributing to the maintenance of stem cell-like properties in the ESCC context (Figure 3). Moreover, the accumulation of D-lactate may induce iron-dependent tumor cells death phenotypes involving elevated ROS levels and intracellular iron content. The starting point of this behavior depends on the phosphorylation of nuclear YAP at the S127 and S397 sites mediated by CDK7, promoting *LDHD* transcription and the subsequent production of pyruvate from D-Lactate catabolism, particularly in mitochondria. Hence, the authors identified the CDK7-YAP-LDHD axis as having the capability to sustain stem cell properties in ESCC, suggesting the possibility of targeting it to improve cancer therapy [33].

### 2.5. Involvement of MAPKs in Ferroptosis Regulation

#### RTKs Control MAPKs Showing Pro- and Anti-Ferroptotic Activation

**Figure 4:**
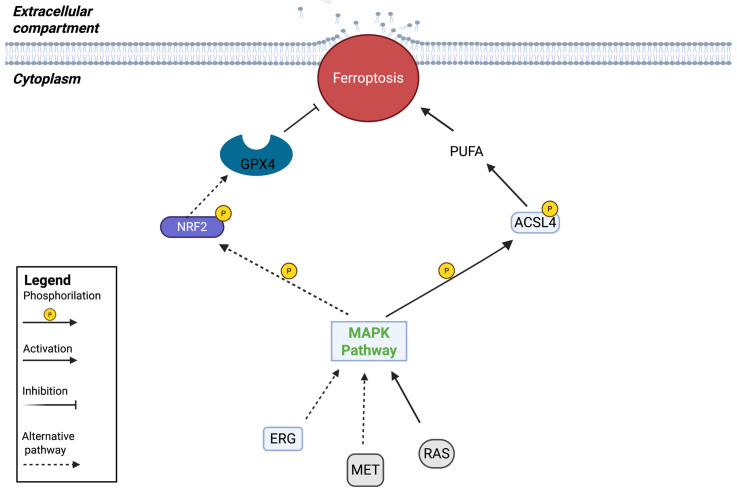
MAPK pathway involved in ferroptosis mechanisms can positively or negatively affect the activation of ferroptosis.

The discovery of the role of the MAPK pathway in ROS production has prompted researchers to study its role in cancer growth. It is now clear that ferroptosis is generally accompanied by the inhibition of MAPK signaling, suggesting that targeting this pathway could be a promising strategy for anticancer therapy by inducing ferroptosis. Some authors have discussed the potential of modulating MAPK signaling to induce ROS generation and, subsequently, ferroptosis. Jin et al. demonstrated the important involvement of receptor tyrosine kinases (RTK) in the ferroptotic pathway, leading to ferroptosis downregulation through the action of MAPK kinases [94]. Indeed, RTKs are able to regulate MAPK kinases in response to specific extracellular stimuli. EGFR and MET are RTKs that promote MAPK signaling, and their activation has pro-tumoral properties [67]. FMS-like tyrosine kinases exhibit a ferroptosis inhibitory function [95]. Nrf2, which has already been shown to play a pivotal role in the mechanism of cancer ferroptosis, could be a downstream target of RTKs [60]. Interestingly, it has been noted that RTK activation can increase cellular susceptibility to ferroptosis rather than inhibiting it. The upregulation of ACSL4, a ferroptosis promoter, can be induced by RTK activation through the RAS/RAF/c-Myc axis, implicating the dual function of RTKs in regulating ferroptosis. Thus, the activity of RTKs is more complex, and their pro- or anti-ferroptosis function (Figure 4) depends on the cellular context [66]. 

## 3. Discussion

The field of ferroptosis is broad and complex. It involves several factors and kinases in all branches of the molecular mechanisms that sustain or repress lipid peroxidation. Researchers typically focus on targets that can make a huge contribution to improving current anticancer therapies, and kinases are no exception. Because several kinases control pathways that can either increase or decrease ferroptosis levels, targeting kinases can control pro- or anti-ferroptotic factors, depending on the pathology, the cell type and the methodology used to fight a disease. Several kinase-dependent mechanisms have been discovered that can support or impair ferroptosis in different ways, providing several possible scenarios for developing the most appropriate and effective therapy. The various pathways that control ferroptosis, which involve numerous factors, act cooperatively or independently in the intricate network that regulates ferroptosis. GPX4 is the main protector against ferroptosis, as it removes free radicals using its cofactor glutathione, through redox activity. By investigating ferroptotic pathways, researchers have found kinase-dependent-control of GPX4 activity, both through direct regulation and indirectly through transcription factors such as Nrf2, which, in turn, can regulate GPX4 expression. However, other mechanisms controlling ferroptosis that do not necessarily involve GPX4 are also controlled by kinases, such as SLC7A11, LDHD and the mTOR pathway. At the same time, pro-ferroptotic factors, ACSL4, for example, have also been studied, and the involvement of kinases in regulating their activity has been demonstrated. Given their capacity to regulate ferroptosis, kinases are inevitably involved in cancer development, where they regulate ferroptosis and play an intriguing role in tumorigenesis by protecting cancer cells from ferroptosis and enabling them to enhance their proliferative capacity. For this reason, having a clear and comprehensive picture of kinase activity in ferroptosis may be important, as it could contribute to a better understanding of how ferroptosis affects cancer and, more importantly, can expand the potential to develop strategies to improve therapy. In the field of cancer therapy, it is well known that one of the most important challenges is overcoming resistance to treatment in tumor cells. Ferroptosis, with its ability to contribute to cancer cell death, may represent a new strategic tool to overcome this obstacle. Given the relatively recent discovery of ferroptosis mechanisms, many aspects still need to be clarified, and the role of kinases is not an exception.

Some kinase inhibitors already authorized for treatment of patients with several pathologies may also be studied in the context of ferroptosis, to validate their use in combination with other compounds. Sorafenib, for example, has been approved for the treatment of HCC, advanced renal cell carcinoma, and differentiated thyroid cancer [62]. Recently, Sorafenib has been shown to trigger ferroptosis in vitro, although this activation may depend on the cellular context. Other tyrosine kinase inhibitors, such as Lapatinib and Neratinib, have been approved for the treatment of breast cancer. Lapatinab has also been linked to the induction of ferroptosis in vitro, indicating that studying the mechanisms used by cells in ferroptosis resistance in depth could be crucial for developing new strategies. The case of Lapatinib is highly indicative because scientists, by delving deeper into its role, found a combination therapy with another compound, Siranesine, which is showing high efficacy [96,97]. Another case concerns Neratinib, which can sensitize breast cancer cells to ferroptosis [98]. Its utility in triggering ferroptosis has been used to design a combined therapy with RSL3, a ferroptosis inducer, in order to obtain a stronger pro-ferroptotic effect [99]. These studies indicated that targeting kinases in the context of ferroptosis may be a valid and useful strategy for fighting cancer, particularly when used in combination therapies. However, already-approved kinase inhibitors have shown some limitations, especially depending on the context, and further studies are needed to provide a clear and acceptable role for these drugs in ferroptotic mechanisms when used in combination therapies [100]. Nevertheless, the fact that these compounds are already approved in some contexts is an important indication of their applicability and safety. This may be a starting point, not only to clarify how already-tested kinase inhibitors can be used but also to begin the investigation of other compounds capable of targeting other kinases involved in ferroptosis.

Through this review, we analyzed various aspects of the role of kinases in the context of ferroptosis in cancer, aiming to show the clearest possible overview of their contribution to pro- and anti-ferroptotic activities. This may provide useful tools for researchers to have a complete scenario from which to develop new strategies. However, these data demonstrate that they must be treated with a critical eye. In fact, focusing on this area has some limitations. First, several data have shown promising results concerning the use of compounds capable of targeting ferroptotic pathways. However, although some kinase inhibitors are already approved, experiments involving kinases that have only recently been associated with ferroptotic pathways are often limited to in vitro studies or in mouse models. Therefore, their actual contribution in humans remains unclear, as does how targeting kinases might improve current therapies such as immunotherapy. Second, although ferroptosis seems to have a strong implication in the cancer context, it is certainly not the sole determinant of cancer growth, which depends on many factors independent of ferroptosis. Thus, targeting ferroptosis by targeting kinases can only partially contribute to cancer treatment, particularly in those cancer types in which ferroptosis is relevant, and this is not true for all tumors and especially not for all tumor stages. Researchers must consider this aspect to specifically focus on the precise timing of intervention. Furthermore, the data still require deeper investigation, in order to choose the most appropriate target for different tumor contexts. In fact, focusing only on the same targets across different tumor contexts may be the wrong choice, and each cancer type must be evaluated on a case-by-case basis, paying attention to the specific contribution of each kinase to ferroptosis.

In summary, however, we believe it is important to expand our knowledge of the roles of kinases in the development of ferroptosis-dependent cancer, since the results obtained are very promising, but research will require time to find the best direction to use these data.

## Figures and Tables

**Table 1:** The table represents kinases involved in ferroptosis pathways in cancer and their mechanisms of action.

Kinases	Direct Target	Mechanism	Cancer Type
PLK1	CBX8	Phosphorylation of CBX8 promotes the activation of GPX4	Colorectal cancer [43]
SGK2	FOXO1	SGK2 expression induced the nuclear exclusion of FOXO1, whose function is to negatively regulate GPX4	Prostate cancer [53]
Src	Nrf2, p62	Src sustains the localization in the nucleus of Nrf2, and can also cause the aggregation of p62 and the colocalization with KEAP1 factor	Glioblastoma [60]
AURKA	KEAP1	FOXM1 transcriptionally activates AURKA expression which phosphorylates KEAP1 permitting Nrf2 activation	Meningioma [63]
TBK1	p62	Phosphorylation of p62 causes aggregation with KEAP1	Hepatocellular carcinoma [55]
CDK7	YAP	The kinase CDK7 phosphorylates YAP which, in turn, may enhance LDHD expression. LDHD is able to accelerate D-lactate catabolism bringing to a protection against ferroptosis	Esophageal cell carcinoma [33]
SIK1	HDAC5	SIK1 phosphorylates HDAC5, which is stabilized. HDAC5 deacetylates *STAT6*, that upregulates SLC7A11	Pancreatic ductal adenocarcinoma [14]
mTORC1	SREBP1	mTOR phosphorylates SREBP1 which regulates SCD1. SCD1 Produces MUFA that can protect from ferroptosis	Breast and prostate cancer [49]
PKCβII	ACSL4	PKCβI directly phosphorylates ACSL4 triggering PUFA-containing lipid biosynthesis and promoting the generation of lipid-peroxidation products	Breast cancer [28]
MAPK	Nrf2	ERG and MET can promote MAPK pathway and the Phosphorylation of Nrf2 induces anti- ferroptotic genes	Breast cancer [67]
MAPK	ACSL4	RTK activation through the RAS/RAF/c-Myc axis phosphorylates ACSL4	Fibrosarcoma [66]

## Data Availability

No new data were created or analyzed in this study. Data sharing is not applicable to this article.

## Abbreviation List

GPX4	Glutathione Peroxidase 4
GSH	Glutathione
ROS	Reactive Oxygen Species
LOX	Lipoxygenases
TF	Transferrin
TFR	Transferrin Receptor
SLC7A11	Solute Carrier Family 7 Member 11
SLC3A2	Solute Carrier Family 3 Member 2
MAPK	Mitogen-Activated Protein Kinase
AMPK	AMP-activated Protein Kinase
ERK	Extracellular signal-Regulated Kinase
JNK	c-Jun N-terminal Kinase
SIK1	Salt-Inducible Kinase 1
PDAC	Pancreatic Ductal Adenocarcinoma
PKCβII	Protein Kinase C family of Ser/Thr kinases
ACSL4	Acyl-CoA Synthetase Long-chain family member 4
CDK	Cyclin-Dependent Kinases
ESCC	Esophageal Squamous Cell Carcinoma
YAP	Yes-Associated Protein
LDHD	Lactate Dehydrogenase D
PLK1	Polo-Like Kinase 1
PBD	Polo-Box Domain
mCRC	Metastatic Colorectal Cancer
EGFR	Epidermal Growth Factor Receptor
PI3K	Phosphatidylinositol 3-kinase
AKT	Ak Strain Transforming
mTOR	mammalian Target Of Rapamycin
PTEN	Phosphatase and TENsin homolog
SREBP-1	Sterol Regulatory Element-Binding Protein-1
SGK	Serum and Glucocorticoid Kinases
TBK1	TANK-binding kinase 1
IKK	the IκB Kinase
IFN	Interferon
CBX8	Chromobox homolog 8
FOXO	Forkhead box, sub-group O
PKB	Protein Kinase B
PC	Prostate Cancer
KEAP1	Kelch-like ECH-associated protein 1
GBM	Glioblastoma
AURKA	Aurora Kinase A
FTH1	Ferritin Heavy chain 1
FTL	Ferritin Light chain
HMOX1	Heme Oxygenase 1
HCC	Hepatocellular Carcinoma
G6PD	Glucose-6-Phosphate Dehydrogenase
PDAC	Pancreatic Ductal Adenocarcinoma
HDAC	Histone Deacetylase
STAT	Signal Transducer and Activator of Transcription
TRIM	Tripartite Motif
SCD1	Stearoyl-CoA desaturase-1
MUFA	Monounsaturated fatty acids
PUFA	Polyunsaturated Fatty Acids
RTK	Receptor Tyrosine Kinases
